# Tirofiban hydrochloride sodium chloride injection combined with cardiovascular intervention in the treatment of Acute Myocardial Infarction

**DOI:** 10.12669/pjms.36.2.1005

**Published:** 2020

**Authors:** Yongxuan Liu, Lingling Zhang, Yanyan Yang

**Affiliations:** 1 Yongxuan Liu, Department of Cardiovascular, Binzhou People’s Hospital, Shandong, 256610, China; 2 Lingling Zhang, Department of Cardiovascular, Binzhou People’s Hospital, Shandong, 256610, China; 3 Yanyan Yang, Department of Pharmacy Intravenous Admixture Service, Binzhou People’s Hospital, Shandong, 256610, China

**Keywords:** Acute myocardial infarction, Adverse cardiac events, Cardiovascular intervention, Tirofiban hydrochloride sodium chloride injection

## Abstract

**Objective:**

To investigate the therapeutic effect of tirofiban hydrochloride sodium chloride injection combined with cardiovascular intervention on acute myocardial infarction.

**Methods:**

Eighty-four patients with acute myocardial infarction who were treated in our hospital from April 2017 to May 2018 were divided into a control group and a treatment group using random number table method; there were 42 patients in each group. Both groups were given conventional treatment and were treated with aspirin and clopidogrel before and after operation. Moreover, the control group was treated with cardiovascular interventional therapy, while the treatment group was treated with tirofiban hydrochloride on the basis of cardiovascular interventional therapy. The patients were followed up to observe and compare the treatment condition and adverse reactions of the two groups.

**Results:**

After percutaneous coronary intervention, the normal rate of myocardial perfusion in the treatment group was 92.86%, which was higher than 69.05% in the control group (P<0.05). After PCI, there were significant differences of thrombolysis in myocardial infarction (TIMI) flow Grade- 2 and 3 between the two groups (P<0.05). The improvement of platelet activation function in the treatment group was better than that in the control group (P<0.05). The incidence of adverse cardiac events in the treatment group was significantly lower than that in the control group (P<0.05).

**Conclusion:**

Tirofiban hydrochloride sodium chloride injection combined with cardiovascular intervention has a significant clinical effect in the treatment of acute myocardial infarction. It can effectively improve the blood perfusion and reduce the incidence of adverse cardiac events, suggesting a good effect on the prognosis of patients and high application value.

## INTRODUCTION

Acute myocardial infarction is a disease which occurs and progresses rapidly and has high mortality rate.^1^-^3^ With the development of social economy and the changes of living habits of people, acute myocardial infarction has been one of the diseases which can threaten public health. Acute ST-segment elevation myocardial infarction is a commonly seen type of infarction.^4^,^5^ It will further induce coronary thrombosis, and the formation of thrombosis will accelerate the release of procoagulant substances and strengthen the coagulation potential of human body.

Currently, cardiovascular interventional therapy is the most commonly used therapy in the treatment of acute myocardial infarction, which can reduce the size of infarction and protect myocardial function.^6^ However, distal embolism may occur in the course of cardiovascular interventional therapy, slowing down the speed of blood perfusion, and no-reflow phenomenon will occur in severe cases, which will lead to adverse cardiovascular events in the prognosis of patients. Therefore, in the treatment of acute myocardial infarction, it is necessary to use effective schemes to inhibit the formation of thrombus.^7^,^8^ Thus, in the treatment of acute myocardial infarction, it is very important to take effective measures to inhibit platelet thrombosis. Aspirin and clopidogrel are commonly used anti-platelet drugs in clinic and can also be used for treating acute coronary syndrome, which can inhibit platelet aggregation and improve the prognosis of patients.^9^ However, aspirin and clopidogrel can only block one way of platelet activation, and platelets can also be activated and aggregated by other ways, which limits their clinical efficacy. In recent years, studies have shown that tirofiban hydrochloride sodium chloride injection can effectively inhibit platelet aggregation and improve patients’ cardiac function index.^10^ In this study, 84 patients with acute myocardial infarction were selected to evaluate the therapeutic effect of tirofiban hydrochloride sodium chloride injection combined with cardiovascular intervention, in order to provide effective reference for clinical rational drug use.

## METHODS

Eighty-four patients with ST-segment elevation myocardial infarction who were admitted to our hospital from April 2017 to May 2018 were selected as the study subjects. They were randomly divided into a treatment group and a control group, 42 cases each. There were 25 males and 17 females in the observation group, and they aged from 43 to 77 years, with an average age of (60.77±3.08) years. There were 24 males and 18 females in the control group, and they aged 42-77 years, with an average age of (60.56±3.17) years. There was no significant difference in general data between the two groups (P>0.05). All patients met the following inclusion criteria: time from the onset to the implementation of PCI < 12 h; conforming to the diagnostic criteria of ST-segment elevation myocardial infarction in Guideline for Diagnosis and Treatment of Patients with ST-elevation Myocardial Infarction,^11^ and being diagnosed as myocardial infarction for the first time. Exclusion criteria included undergoing coronary artery bypass grafting within one month, having malignant arrhythmia and severe heart, brain, lung and kidney dysfunction, being allergic to tirofiban hydrochloride or with anticoagulant contraindications, and being pregnant or breast-feeding. This study was approved by the medical ethics committee of our hospital. All patients signed the informed consent.

### Therapeutic method

Both groups received conventional treatment, including resting in bed and oxygen uptake. After the family members of the patients signed informed consent, the condition of artery was checked by performing coronary angiography. Each patient orally took aspirin enteric-coated tablets at the dose of 300 mg and clopidogrel tablets at the dose of 300 mg two hours before surgery. After operation, each patient orally took clopidogrel at the dose of 75 mg·d^-1^ and aspirin enteric-coated tablets at the dose of 100 mg·d^-1^. Heparin sodium injection was injected at the dose of 100 U·kg^-1^ via the side tube of sheathing canal during PCI. The treatment group was intravenously injected with tirofiban hydrochloride 30 minutes before PCI; 5 min before PCI, it was intravenously injected at a dose of 10 μg·kg^-1^, and then it was injected for 24 h ~ 36 h to maintain the plasma drug concentration at 0. 15 μg·kg^-1^·minutes^-1^. After PCI, patients in the two groups were subcutaneously injected with 5000 U low-molecular-weight heparin injection, once every 12 h, three and five days each time.

### Observation indicators and evaluation criteria

(1) TIMI Myocardial Perfusion Grading (TMPG) was performed within 15 minutes after percutaneous coronary intervention (PCI): grade 0 indicates that myocardial perfusion is not obvious; grade I indicates that the perfusion of contrast medium is slow; grade II indicates that there is a delay in contrast medium into and out of micro vessels; grade III indicates that contrast medium can be perfused effectively. The normal rate of myocardial perfusion = TMPG Grade-III/n × 100%. The immediate post-surgical thrombolysis in myocardial infarction (TIMI) flow was compared between the two groups. TIMI grade 0 indicated no blood perfusion at the closed distant vessel; TIMI Grade-I indicated forward flow was difficult to fill the distant vascular bed of the lesion; TIMI Grade-II indicated the distant vascular bed of the lesion could be effectively filled after more than three cardiac cycles; TIMI Grade-III indicated that the distant vessel could be completely filled within three cardiac cycles. Platelet activation functions of the two groups before and after treatment were recorded, including positive expression rates of platelet membrane glycoprotein (CD62P), liposomal glycoprotein (CD63) and monocyte platelet aggregation (MPA). Detection of platelet activity function was as follows. three mL of peripheral venous blood was collected before and after treatment, and monoclonal immunofluorescent antibody was detected. The positive expression of CD62P, CD63 and MPA was detected by Quest software. Adverse cardiac events of the two groups were recorded and compared.

### Statistical analysis

SPSS20.0 was used for data processing and analysis. Measurement data were expressed as Mean±SD. Mean comparison was performed by t test. Counting data were compared by X^2^ test (general data) or rank sum test (rank data). When P<0.05, the difference has statistical significance.

## RESULTS

### Comparison of TMPG grade between the two groups

After PCI, the normal rate of myocardial perfusion was 92.86% in the treatment group, which was significantly higher than 69.05% in the control group (P<0.05 Table-I).

**Table-I:** Comparison of TMPG grade after PCI.

Group	Treatment group	Control group	X^2^	P
Grade-I	0	4	/	/
Grade-II	3	9		
Grade-III	39	29		
Normal rate of myocardial perfusion (%)	92.86	69.05	6.178	<0.05

### Comparison of TIMI flow between the two groups after PCI

After PCI, patients with TIMI Grade-II in the treatment group were less than those in the control group, and patients with TIMI Grade-III were more than those in the control group; the differences were statistically significant (P < 0.05, Table-II).

**Table-II:** Comparison of TIMI grade after PCI.

Group	Treatment group	Control group
Grade-I	1	3
Grade-II	2	10
Grade-III	39	29

***Note:*** In the comparison between the two groups, Uc=3.261, P=0.001.

### Comparison of platelet activation function between the two groups

The positive expression rates of CD62P, CD63 and MPA in the two groups before treatment were not significantly different (P>0.05); after treatment, the positive expression rates of CD62P, CD63 and MPA in the two groups were improved, and the positive expression rates of CD62P, CD63 and MPA in the treatment group were significantly lower than those in the control group (P<0.05, Table-III).

**Table-III:** Positive expression rates of CD62P, CD63 and MPA before and after treatment (%, Mean±SD).

Group	Treatment group		Control group	

	Before treatment	After treatment	Before treatment	After treatment
CD62P	7.26±1.15	1.79±0.67*#	7.21±1.18	2.89±0.86*
CD63	6.85±1.16	1.68±0.16*#	6.98±1.06	3.96±1.12*
MPA	21.32±5.66	11.47±1.45*#	21.18±5.26	16.96±2.27*

***Note:***

* means P<0.05 compared to before treatment, #means P<0.05 compared to the control group after treatment.

### Comparison of adverse cardiac events between the two groups after operation

The incidence of adverse cardiac events in the treatment group was significantly lower than that in the control group, and the difference had statistical significance (P<0.05, Table-IV).

**Table-IV:** Adverse cardiac events between the two groups (%).

Group	Treatment group	Control group	X^2^	P
Revascularization	1(2.38)	2(4.76)	/	/
Malignant arrhythmia	0(0)	2(4.76)		
Refractory myocardial ischemia	1(2.38)	3(7.14)		
Death	0(0)	1(2.38)		
Incidence of adverse cardiac events	2(4.76)	8(19.04)	5.236	<0.05

## DISCUSSION

Acute myocardial infarction is a cardiovascular disease with a high clinical incidence. It has a rapid onset, rapid development, high mortality and disability rate, which poses a serious threat to patients’ life safety and quality of life.^12^ Stent implantation, is the most effective treatment for ST-segment elevation myocardial infarction currently according to the foreign treatment guidelines.^13^ However, PCI can damage vascular endothelium, promote platelet activation and adhesion, and induce thrombus. As study has shown that the occurrence of cardiovascular events after PCI is closely related to platelets.^14^ Although PCI can effectively recanalize the coronary artery, sometimes myocardial reperfusion is not successful; therefore, ant platelet therapy is not only the key to the treatment of ST-segment elevation myocardial infarction, but also an important part of preventing cardiovascular events during and after PCI.

Tirofiban hydrochloride is a kind of the third generation of platelet membrane receptor antagonist, which is highly efficient and irreversibly in inhibiting platelet adhesion and aggregation.^15^ The study of Liu^16^ demonstrates that tirofiban hydrochloride can promote the improvement of prognosis on the basis of improving blood perfusion after treatment for patients with acute myocardial infarction. Moreover another study^17^ found that the application of tirofiban hydrochloride could increase the postoperative bleeding events after treatment for patients with acute myocardial infarction. In this study, there was only few cases of bleeding, which might be related to the small sample size.

Moreover the results of this study demonstrated that the TMPG of the treatment group was better than that of the control group, which was because that tirofiban could inhibit the contraction of vascular substances and reduce the release of inflammatory factors, thus improving the recovery of arterial blood flow and myocardial microcirculation.^18^ In addition, this study also discussed the changes of activation function indexes of patients before and after treatment. The results showed that the positive expression rates of CD62P, CD63 and MPA in the treatment group after treatment were significantly better than those before treatment and in the control group, suggesting that adding tirofiban hydrochloride sodium chloride injection on the basis of vascular interventional therapy could achieve a better therapeutic effect and improve platelet activation function. The results of this study are similar to those of related studies.^19^,^20^

After vascular interventional therapy, patients have many adverse reactions due to inflammation and activation of platelet in atherosclerotic plaques after interventional therapy, resulting in poor prognosis of patients.^21^ The combined use of tirofiban and vascular interventional therapy can effectively improve adverse reactions and prognosis; the reason is that tirofiban has a good anti-platelet aggregation effect, and moreover the incidence of cardiovascular events is significantly reduced because of the increased platelet activation and reduced thrombosis. In the comparison of adverse reactions between the two groups, the results showed that the incidence of adverse cardiac events in the treatment group was smaller than that in the control group, and the difference was statistically significant (P<0.05). These results were similar to the results of Nie.^22^

## CONCLUSION

Tirofiban hydrochloride sodium chloride injection combined with cardiovascular interventional therapy can effectively improve the clinical efficacy and prognosis of patients with acute myocardial infarction, which has high clinical value and is worth promotion and implementation. But the sample size of this study was small and the observation time was short; in the future study, we will further expand the sample size, improve the research process and methods, and evaluate the clinical effect and safety of tirofiban hydrochloride from various perspectives, so as to provide reference for practical clinical application.

### Authors’ Contribution:

**YXL:** Study design, data collection and analysis, takes responsibility for integrity of research.

**YXL, LLZ & YYY:** Manuscript preparation, drafting and revising.

**YXL:** Review and final approval of manuscript.
